# Eignung der Bestimmung prozentualer Hörverluste zum Monitoring der Cochlea Implantat Rehabilitation

**DOI:** 10.1007/s00106-022-01257-8

**Published:** 2023-01-02

**Authors:** Oliver C. Dziemba, Tina Brzoska, Friedrich Ihler, Chia-Jung Busch

**Affiliations:** Klinik und Poliklinik für Hals‑, Nasen‑, Ohrenkrankheiten, Kopf- und Halschirurgie, Universitätsmedizin Greifswald, Ferdinand-Sauerbruch-Str., 17475 Greifswald, Deutschland

**Keywords:** Cochleaimplantat, Lautheitsskalierung, Sprachaudiometrie, Rehabilitation, Qualitätskontrolle, Cochlear implant, Loudness scaling, Speech audiometry, Rehabilitation, Quality control

## Abstract

**Hintergrund:**

Die Bestimmung des prozentualen Hörverlusts (pHV) aus dem Tonaudiogramm nach Röser 1973 oder aus dem Sprachaudiogramm nach Boenninghaus und Röser 1973 sind weithin eingesetzte Methoden zur quantitativen Bemessung des Hörvermögens. Die Bestimmung von pHV aus ton- und sprachaudiometrischen Befunden im Rahmen der postoperativen Evaluation von Hörsystemen ist bisher nicht üblich. Bei Durchführung aller empfohlenen audiologischen Leistungen nach der Indikation für ein Cochleaimplantat (CI) liegen alle nötigen Messwerte zur Bestimmung des pHV aus der Hörfeldskalierung (pHV_KLS_) und aus dem Sprachaudiogramm (pHV_FB_) vor.

**Ziel der Arbeit:**

Die Parameter pHV_KLS_ and pVH_FB_ sollen vorgestellt und anhand von Daten aus der klinischen Routine berechnet werden. Dadurch soll die prinzipielle Verwendbarkeit für die Bewertung des Ergebnisses einer CI-Versorgung evaluiert werden.

**Material und Methoden:**

Retrospektive Auswertung der Daten von 66 CI-Versorgungen an einer Universitätsklinik. Kalkulation des prozentualen Hörverlusts pHV aus dem numerischen Skalenwert 5 CU der kategorialen Lautskalierung (pHV_KLS_) bzw. aus dem Freiburger Sprachtest im freien Schallfeld mit CI (pHV_FB_).

**Ergebnisse:**

Während die Werte des pHV_KLS_ eine geringe Streuung aufweisen, zeigt pHV_FB_ eine größere, aber über der Zeit abnehmende Streuung. Außerdem zeigt sich eine Konvergenz der mittleren pHV ab dem Zeitpunkt der CI-Erstanpassung. Die Differenz aus pHV_FB_ und pHV_KLS_ ergibt mit statistischer Signifikanz positive Werte.

**Schlussfolgerung:**

Die Bestimmung des pHV aus der kategorialen Lautheitsskalierung bzw. aus dem Freiburger Sprachtest ist in der klinischen Routine möglich. Eine Korrelation der Differenz aus pHV_FB_ und pHV_KLS_ mit dem Erfolg der CI-Versorgung erscheint plausibel.

## Abstract (en)

**Background:**

Calculation of percentage hearing loss (pHV) from the pure-tune audiogram according to Röser in 1973 or from the speech audiogram according to Boenninghaus and Röser in 1973 is a method still applied for quantitative assessment of hearing. However, this is not common for the evaluation of postoperative results of implantable hearing systems. During the regular work-up after cochlear implantation (CI) in Germany, all necessary parameters are available for calculation of pHV either from categorical loudness scaling (pHV_KLS_) or speech-recognition threshold (pHV_FB_).

**Objective:**

The parameters pHV_KLS_ and pHV_FB_ are introduced and calculated from data available from clinical routine. Their potential applicability for assessment of the result of CI is evaluated.

**Materials and methods:**

This study comprises retrospective chart review of audiological parameters from 66 CI procedures in one tertiary referral center. pHV_KLS_ was calculated from the equal loudness curve 5 CU, pHV_FB_ from the Freiburg speech test in free field.

**Results:**

While pHV_KLS_ shows small variation, the variation in pHV_FB_ is initially larger but decreases over time. Furthermore, starting from initial fitting, the mean pHV shows convergence over time. The difference between pHV_FB_ and pHV_KLS_ is positive and statistically significant.

**Conclusion:**

It is possible to calculate pHV_KLS_ and pHV_FB_ from routine data. A correlation of the difference between pHV_FB_ and pHV_KLS_ with successful CI performance seems plausible.

## Einleitung

Mit der Ausdehnung der Entschädigungspflicht der Sozialversicherung auf bestimmte gewerblich bedingte Ohrenleiden, auf durch Lärm verursachte Taubheit oder an Taubheit grenzende Schwerhörigkeit im Jahr 1929 [16] wuchsen die Anforderungen an die gutachterliche audiologische Hörprüfung stetig an. Im Rahmen solcher audiologischer Hörprüfungen können durch verschiedene ton- und sprachaudiometrische Messverfahren zahlreiche Messwerte erhoben werden, die in anschließender Zusammenschau eine differenzierte Diagnostik und Begutachtung einer vorliegenden Hörstörung erlauben. Auf die Einführung der apparativen Sprachaudiometrie [11] in der Mitte des vorigen Jahrhunderts folgten unter anderem Bestrebungen, die einzelnen Messwerte der Sprachaudiometrie in einem Zahlenwert zu konzentrieren und so den prozentualen Hörverlust (pHV) gegenüber Normalhörenden darstellen zu können [3]. Diese Methode der Bestimmung des pHV aus dem Sprachaudiogramm wurde von Boenninghaus und Röser [3, 4] vorgestellt, von Feldmann [9] verbessert und schließlich von Brusis [5] überarbeitet [4]. Diese Methode wird bis heute bei der quantitativen Bemessung des Hörvermögens, beispielsweise bei der Begutachtung von Schwerhörigkeiten, angewendet [10]. Vor dem gleichen Hintergrund stellte Röser [20, 21] verschiedene Tabellen zur Schätzung des pHV aus dem Tonaudiogramm vor.

Bei der Entwicklung der verschiedenen Methoden zur Bestimmung des pHV wurde zunächst darauf geachtet, dass eine vorliegende Schwerhörigkeit unabhängig von der verwendeten Methode einen möglichst ähnlichen Wert annahm. Dieser Ansatz kann bei der Bewertung eines rehabilitativen Versorgungsprozesses, wie beispielsweise bei der Cochleaimplantat(CI)-Versorgung, nicht vorausgesetzt werden. Nach Annahme der Autoren kann die Entwicklung der Sprachverständlichkeit im Rahmen der CI-Rehabilitation nur bei optimaler Einstellung der Stimulationsparameter das gewünschte Versorgungsziel erreichen. So konnten zum Beispiel Rader et al. [18] zeigen, dass die Optimierung der Parameter zur CI-Stimulation insbesondere auf dem Niveau der Wahrnehmbarkeitsschwelle der Elektrostimulation die Basis einer verbesserten Sprachverständlichkeit mit CI darstellt. Die Autoren leiten daraus die weitere Annahme ab, dass sich die Wirkung eines multidisziplinären CI-Rehabilitationskonzepts in der Konvergenz des pHV über der Zeit der Hörerfahrung, ermittelt aus ton- und sprachaudiometrischen Befunden, zeigen muss.

Ziel dieser Arbeit ist es, durch retrospektive Analyse der verfügbaren ton- und sprachaudiometrischen Daten aus dem CI-Versorgungsprozess an der Einrichtung der Autoren die Eignung der Bestimmung der pHV aus ton- und sprachaudiometrischen Befunden über der Zeit zu analysieren und die Möglichkeiten zur Evaluation von CI-Versorgungsergebnissen zu prüfen. Die Grundannahme besteht darin, dass sich eine fortschreitende Hörrehabilitation in einer Konvergenz des prozentualen Hörverlusts, bestimmt aus der Sprachaudiometrie mit CI (pHV_FB_), an den prozentualen Hörverlust, bestimmt aus der kategorialen Lautheitsskalierung mit CI (pHV_KLS_), zeigt. Darüber hinaus sollen die drei verschiedenen Ansätze zur Bestimmung des pHV aus dem Sprachaudiogramm [3–5, 9] gegenüber gestellt werden.

## Material und Methoden

### Bestimmung des prozentualen Hörverlusts mit CI aus der Sprachaudiometrie

Das von Boenninghaus und Röser vorgestellte und modifizierte Verfahren [3, 4] zur Bestimmung des pHV aus dem Freiburger Sprachaudiogramm [11] verwendet den Hörverlust für Zahlen (HVZ), auch als a_1_-Wert bezeichnet, und das Gesamtwortverstehen (w_S_). Der HVZ ergibt sich aus der Differenz der 50%igen Sprachverständlichkeit für Zahlwörter des gemessenen Werts und dem Pegel der Referenzkurve bei 50 % (18,4 dB_SPL_). Das Gesamtwortverstehen berechnet sich aus der Summe der prozentualen Sprachverständlichkeit bei 60 dB_SPL_, 80 dB_SPL_, 100 dB_SPL_. Alternativ kann das gewichtete Gesamtwortverstehen berechnet werden [9]. Dabei werden die prozentualen Sprachverständlichkeitswerte gewichtet (3fach bei 60 dB_SPL_, 2fach bei 80 dB_SPL_, einfach bei 100 dB_SPL_), aufsummiert und die Summe durch zwei dividiert. Aus der Tabelle zur Ermittlung des pHV aus den Werten der Sprachaudiometrie nach Boenninghaus und Röser [4] kann nun der pHV abgelesen werden.

Die von Brusis [5] vorgeschlagene Vereinfachung der Berechnung des pHV aus dem Sprachaudiogramm zielte auf eine genauere Abbildung der beginnenden und geringgradigen Schwerhörigkeit sowie eine bessere Korrelation mit dem Tonaudiogramm. Dabei wird der pHV mithilfe des HVZ und des w_S_ aus einer überarbeiteten Tabelle bestimmt. Eine Bestimmung des gewichteten w_S_ ist hier nicht notwendig.

Die von Boenninghaus und Röser [4] vorgeschlagenen Pegel zur Ermittlung des w_S_ orientieren sich an den Normalwerten der Einsilberdiskrimination und an der angenommenen Obergrenze für praktisch vorkommende Sprachschallpegel. Sie beschreiben weiter: „[…], man hätte ebenso 55-75-95 dB oder auch 65-85-105 dB ansetzen können, ohne daß [sic!] sich das Ergebnis über die statistischen Grenzen hinaus verändert hätte […]“ [4].

Aufgrund der erforderlichen Dynamikkompression im Rahmen der CI-Vorverarbeitung [15] muss bei CI-Systemen eine deutlich geringere Dynamik um den mittleren Sprachschallpegel von 65 dB_SPL_ angenommen werden. Daher setzen die Autoren für die Ermittlung des gewichteten Gesamtwortverstehens (w_S_) die Sprachschallpegel 50-65-80 dB_SPL_ an und bestimmen den prozentualen Hörverlust aus dem Freiburger Sprachtest im freien Schallfeld mit CI (pHV_FB_) weiter in der oben beschrieben Weise.

### Bestimmung des prozentualen Hörverlusts mit CI aus der Hörfeldskalierung

Zur Bestimmung des pHV aus dem Tonaudiogramm wurden von Röser mehrere Tabellen vorgestellt [19, 20]. Besonders zur Bemessung des Schwerhörigkeitsgrads aus unregelmäßig verlaufenden Tonhörkurven und bei fehlenden Sprachtestmöglichkeiten schlägt Röser die Verwendung einer Vier-Frequenz-Tabelle vor [20]. Der pHV ergibt sich so aus der Addition der Teilkomponenten für die Frequenzen 500 Hz, 1000 Hz, 2000 Hz und 4000 Hz.

Im Rahmen der von der Deutschen Gesellschaft für Audiologie e. V. empfohlenen audiologischen Leistungen nach der CI-Indikation [6] ist eine Hörfeldskalierung bei mindestens den oben genannten Frequenzen obligat. Aus der Hörfeldskalierung können durch Regression Kurven gleicher Lautheit (Isophone) für den gemessenen Frequenzbereich ermittelt werden. Am gebräuchlichsten ist die Darstellung der Isophone für die Lautheitskategorien einer 11-stufigen Skala in sehr leise (5 CU, „categorial unit“, kategoriale Einheit), leise (15 CU), mittel (25 CU), laut (35 CU), sehr laut (45 CU) und zu laut (50 CU) nach DIN ISO 16832 [8]. Für die nicht näher bezeichneten Zwischenstufen werden in der Regel keine Isophone berechnet.

Unter der Annahme, dass eine tonaudiometrisch ermittelte Hörschwelle das *Minimum audibile* darstellt, verwenden die Autoren den numerischen Skalenwert 5 CU der kategorialen Lautskalierung (KLS) nach [8] zur Ermittlung des prozentualen Hörverlusts (pHV_KLS_). Mit den so ermittelten und auf ganzzahlige Werte abgerundeten Pegelwerten wurden die Teilkomponenten zur Ermittlung des pHV_KLS_ aus der Tabelle gemäß Röser 1973 abgelesen.

### Patienten

In diese Untersuchung wurden alle erwachsenen Patienten eingeschlossen, die im Zeitraum 01.01.2016 bis 31.12.2021 an der Einrichtung der Autoren mit CI versorgt wurden und sich mit der anonymisierten Nutzung ihrer Daten aus den klinischen Routinemessungen einverstanden erklärt haben. Patienten, deren Muttersprache nicht Deutsch war, wurden ausgeschlossen. Die Messdaten wurden sowohl präoperativ als auch zum Zeitpunkt der Erstanpassung und 3, 6, 12, 18 sowie 24 Monate nach Erstanpassung erhoben. Die Ermittlung der präoperativen pHV erfolgte definitionsgemäß aus den unversorgten ton- und sprachaudiometrischen Befunden. Die postoperativen pHV wurde wie beschrieben aus der monauralen KLS und dem monauralen Freiburger Sprachtest im freien Schallfeld ermittelt. Eine eventuell nötige Vertäubung der Gegenseite erfolgte bei der KLS durch passive Vertäubung mittels Ohrstöpsel und Kapselgehörschutz und beim Freiburger Sprachtest durch geeignete aktive Vertäubung.

Eine Selektion der Patienten hinsichtlich Ätiologie, Geschlecht, versorgter Seite, Hersteller, Implantat etc. erfolgte nicht. Wenn einzelne Datensätze unvollständig vorlagen, entweder aufgrund unvollständiger Messungen oder nicht durchführbarer Evaluation, wurden diese mit N/A bewertet. Es erfolgte keine Imputation. Bilateral versorge Patienten wurden seitengetrennt in die Analyse aufgenommen.

Insgesamt wurden 66 CI-Versorgungen eingeschlossen. Das mittlere Alter der Patienten zum Zeitpunkt der Operation betrug 60,2 Jahre mit einer Altersspanne von 26 Jahren bis 84 Jahren. An der Einrichtung der Autoren werden CI-Systeme der Hersteller Advanced Bionics (Stäfa, Schweiz), Cochlear (Syndey, Australien) und MED-EL (Innsbruck, Österreich) implantiert. Zur genauen Spezifikation der individuellen CI-Versorgungen wurde eine Übersicht in Tab. 1 erstellt.

**Tab. 1:** Übersicht zur Charakteristik von Implantaten und Patienten

	Implantate	Patienten
*Anzahl*	66	54
*Geschlecht*		
Männlich	N/A	22
Weiblich		32
*Seite*		
Links	34	N/A
Rechts	32	
*Versorgung*		
Einseitig	54	43
Bilateral sequenziell	11	11
Reimplantation	1	1
*Elektrodenträger*		
Contour Advance	9	N/A
Slim Modiolar	15	
Slim Straight	19	
Medium	1	
Flex28	14	
Standard	2	
Mid Scala	6	
*CI-Typ*		
CI512	8	N/A
CI522	8	
CI532	11	
CI612	1	
CI622	11	
CI632	4	
Synchrony	13	
Synchrony2	4	
HiRes 90K	4	
HiRes Ultra 3D	2	
*Hersteller*		
Cochlear	43	N/A
MED-EL	17	
Advanced Bionics	6	

Für die Durchführung der Studie lag ein positives Votum der Ethikkommission der Universitätsmedizin Greifswald mit der Identifikationsnummer BB 049/17 vor.

## Ergebnisse

**Abb. 1:**
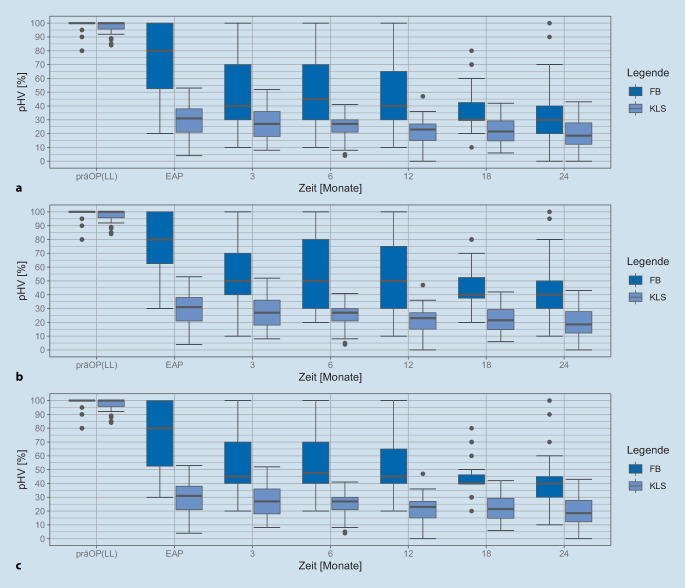
Darstellung der Lage und Streumaße des prozentualen Hörverlusts CI-versorgter Patienten aus der Hörfeldskalierung nach Röser [20] (pHV_KLS_) und [4] aus dem Sprachaudiogramm (pHV_FB_) über die Zeit. **a** Bestimmung des pHV_FB_ nach Boenninghaus und Röser [4] aus HVZ und w_S_. **b** Bestimmung des pHV_FB_ nach Boenninghaus und Röser [4] aus HVZ und gewichtetem w_S_ nach Feldmann [9]. **c** Bestimmung des pHV_FB_ nach Brusis [5] aus HVZ und w_S_. Die *Boxen* zeigen den Median, das erste und das dritte Quartil. Die Whisker zeigen den 1,5fachen Interquartilsabstand. Ausreißer sind als *Punkte* dargestellt

In Abb. 1 sind pHV_KL_ und pHV_FB_ aller verfügbaren Datensätze über den diskreten Zeitpunkten der Routinekontrollen als Lage- und Streumaße dargestellt. In Tab. 2 sind die Anzahlen der ermittelbaren pHV zu den jeweiligen Zeitpunkten aufgelistet. Während die Werte des pHV_KLS_ eine vergleichsweise kleine Streuung von ca. 15 Prozentpunkten aufzeigen, findet sich bei den Werten des pHV_FB_ eine deutlich größere und über der Zeit abnehmende Streuung. Außerdem zeigt sich eine Konvergenz der mittleren pHV ab dem Zeitpunkt der CI-Erstanpassung (EAP).

**Tab. 2:** Anzahlen der ermittelten pHV zu den jeweiligen Zeitpunkten

	präOP(LL)	EAP	3 Monate	6 Monate	12 Monate	18 Monate	24 Monate
pHV_FB_	38	43	30	31	32	35	33
pHV_KLS_	40	33	24	30	34	33	36

*präOP(LL)* präoperative Luftleitung, *EAP* Erstanpassung

**Abb. 2:**
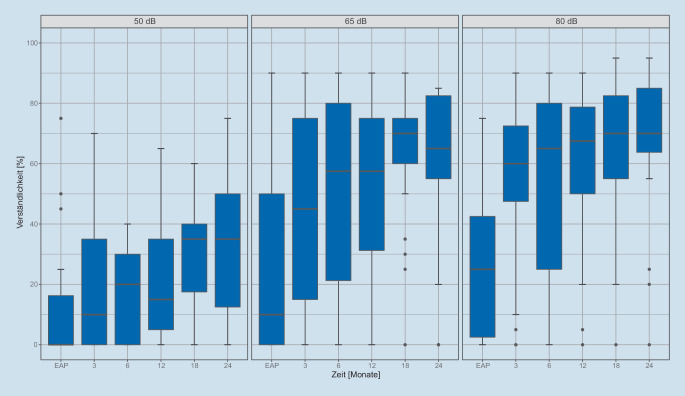
Freiburger Einsilberdiskrimination im freien Schallfeld bei 50-65-80 dB_SPL_ mit CI über die Zeit. Die *Boxen* zeigen Median, erstes und drittes Quartil, die Whisker den 1,5fachen Interquartilsabstand zu den Quartilen an. Ausreißer sind als *Punkte* dargestellt

Die sprachaudiometrischen Ergebnisse im Verlauf der Zeit sind in Abb. 2 und 3 dargestellt. Die Ausreißer in Abb. 2 zeigen hier Nichtnutzer bzw. Fälle mit ausgebliebenem Therapieerfolg.

**Abb. 3:**
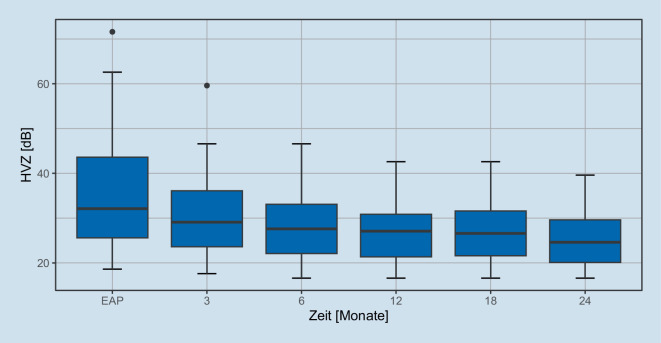
Hörverlust für Zahlen im Freiburger Sprachtest mit CI über die Zeit. Die *Boxen* zeigen Median, erstes und drittes Quartil, die Whisker den 1,5fachen Interquartilsabstand zu den Quartilen an. Ausreißer sind als *Punkte* dargestellt

Unter der Annahme, dass sich eine optimale Sprachverständlichkeit nur auf Basis einer optimalen Hörschwelle einstellen kann, erwarten die Autoren den pHV_FB_ regelhaft größer als den pHV_KLS_. In einem Streudiagramm mit pHV_KLS_ auf der Abszisse und pHV_FB_ auf der Ordinate würden sich die Wertepaare somit links oberhalb der Winkelhalbierenden darstellen.

**Abb. 4:**
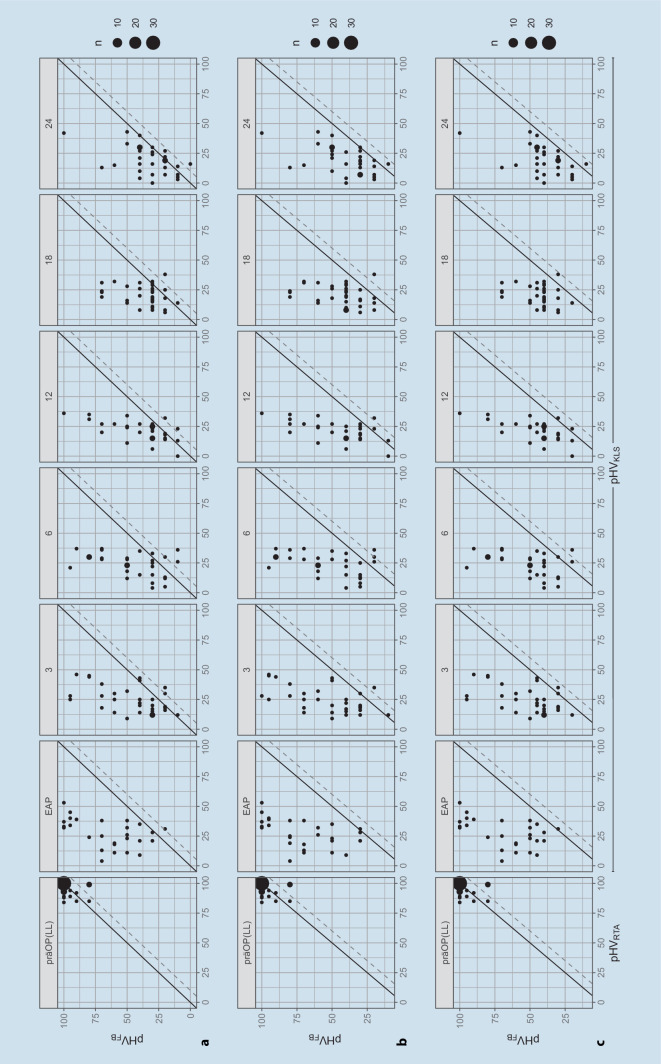
Streudiagramme der pHV zu den verschiedenen Versorgungszeitpunkten. Auf der Abszisse sind pHV aus der Lautheitsskalierung adaptiert nach Röser [20], auf der Ordinate die pHV aus der Sprachaudiometrie [4] dargestellt. **a** Bestimmung des pHV_FB_ nach Boenninghaus und Röser [4] aus HVZ und w_S_. **b** Bestimmung des pHV_FB_ nach Boenninghaus und Röser [4] aus HVZ und gewichtetem w_S_ nach Feldmann [9]. **c** Bestimmung des pHV_FB_ nach Brusis [5] aus HVZ und w_S_. Die *schwarze diagonale Linie* zeigt die Winkelhalbierende, die *graue Strichlinie* die Grenze eines Unsicherheitsbereichs des pHV_KLS_ von 10 dB

In Abb. 4 sind der pHV_FB_ über dem pHV_KLS_ zu den jeweiligen Versorgungszeitpunkten im Streudiagramm dargestellt. Der Durchmesser der einzelnen Punkte symbolisiert dabei das gehäufte Auftreten von Wertepaaren.

Entsprechend der Grundannahme der Autoren, dass die Differenz aus pHV_FB_ und pHV_KLS_ positive Werte annehmen wird, erfolgte ein Test auf Binomialverteilung aller Wertepaare zu den jeweiligen Versorgungszeitpunkten mit der Software R [17, 22]. Fehlende oder unvollständige Werte wurden ausgeschlossen. Die Ergebnisse der Tests auf Binomialverteilung sind in Tab. 3 gelistet. Die Grundannahme der Autoren kann hier als bestätigt interpretiert werden, da die Erfolgswahrscheinlichkeit zu jedem Versorgungszeitpunkt größer als 83 % ist. Diese Aussage kann sogar noch verstärkt werden, wenn eine Ungenauigkeit bei der Bestimmung des pHV_KLS_ von 10 % eingerechnet wird. In diesem Fall liegen die Erfolgswahrscheinlichkeiten des Tests auf Binomialverteilung zu allen Zeitpunkten oberhalb von 92 %.

**Tab. 3:** Ergebnisse des Binomialtests

Zeitpunkt	Anzahl *n*	*p*-Wert	95 %-Konfidenzintervall (%)		Erfolgswahrscheinlichkeit (%)
EAP	26	8,05e-07	80,4	99,9	96,2
3	30	0,00032	65,3	94,4	83,3
6	28	0,00018	67,3	96,0	85,7
12	27	4,93e-05	70,8	97,6	88,9
18	28	2,74e-05	71,8	97,7	89,3
24	29	1,52e-05	72,6	97,8	89,7

*EAP* Erstanpassung

## Diskussion

Die Ermittlung prozentualer Hörverluste aus ton- und/oder sprachaudiometrischen Befunden ist ein etabliertes Verfahren im Rahmen der Begutachtung und der quantitativen Bemessung des menschlichen Hörvermögens. Zur Evaluation vor Hörsystemversorgungen, wie beispielsweise der CI-Versorgung, ist dieses Verfahren bisher nicht eingesetzt worden.

Die Bestimmung des pHV aus den Ergebnissen der kategorialen Lautheitsskalierung ist ohne Weiteres möglich. Hierfür kann die mittels linearer Regression bestimmte Isophone der Kategorie „sehr leise“ verwendet werden und der pHV_KLS_ aus den Teilkomponenten der Tabelle nach Röser [20] aufsummiert werden. Die Methode zur Hörschwellenbestimmung aus der kategorialen Lautheitsskalierung nach Rader et al. [18] könnte alternativ eingesetzt werden. Dieser Ansatz bliebe sogar unabhängig von der Steilheit der einzelnen Pegel-Lautheits-Funktion, setzt jedoch eine hinreichende Genauigkeit des Algorithmus zur adaptiven Pegelsteuerung in Schwellennähe voraus.

Die Bestimmung des pHV aus den sprachaudiometrischen Prüfungen mit dem Freiburger Sprachtest im freien Schallfeld ist ebenso möglich. Bei der Bestimmung des gewichteten Gesamtwortverstehens w_S_ können, unter Berücksichtigung der eingeschränkten überschwelligen Dynamik der proprietären CI-Stimulationsstrategien, die Ergebnisse bei den empfohlenen Messpegeln 50-65-80 dB_SPL_ [6] herangezogen werden.

Zur Vereinfachung der Berechnung des prozentualen Hörverlusts aus dem Sprachaudiogramm und verbesserten Bewertung geringgradiger Hörverluste wurde von Brusis [5] eine überarbeitete Tabelle vorgestellt. Diese Tabelle findet bei der Begutachtung von Lärmschwerhörigkeiten seither Anwendung [7]. Allerdings weisen CI-Träger*innen durch die herstellerspezifische Eingangspegelbegrenzung einen systembedingten Hörverlust auf, der einen milden bis moderaten Hörverlust erwarten lässt [23]. Aus Sicht der Autoren wäre eine gezielte Überarbeitung der Tabellen zur Ermittlung von pHV_KLS_ und pHV_FB_ für eine optimale Bewertung von Hörsystemversorgungen nach dem Vorbild von Brusis wünschenswert.

Die vorgestellten Ergebnisse zeigen, wie erwartet, eine Konvergenz der pHV im Verlauf des Rehabilitationsprozesses. Die Entwicklung der Sprachverständlichkeit mit CI über den Versorgungszeitraum kann sehr individuell verlaufen [2, 14]. Eventuelle Defizite in der schwellennahen Wahrnehmung akustischer Stimuli mit CI können durch präzises Einstellen der CI-Stimulationsparameter im Rahmen des CI-Rehabilitationsprozesses frühzeitig behoben werden [18]. Der pHV_KLS_ bietet hier eine elegante Möglichkeit, die Güte der schwellennahen CI-Einstellung mit nur einem Wert darzustellen. Im Hinblick auf das CI-Versorgungsziel eines bestmöglichen Ausgleichs kann hier der Bezug zur Normalhörigkeit hergestellt werden. In gleicher Weise kann die Entwicklung der Sprachverständlichkeit über der Zeit in nur einem Wert dargestellt werden, da die Ermittlung des pHV_FB_ sowohl die Sprachverständlichkeitsschwelle in Ruhe (HVZ) als auch die gesamte Einsilberdiskrimination in Ruhe über die Bestimmung des Gesamtwortverstehens integriert. Bei Verwendung des gewichteten Gesamtwortverstehens geht die Sprachverständlichkeit bei niedrigen Pegeln mit höherem Gewicht ein. Die Effekte von verbesserten Fitting-Strategien [18] oder verbesserter Mikrofontechnologie bei Prozessorneuversorgungen [12] würden sich so deutlich im pHV_FB_ zeigen.

Die individuelle Entwicklung der Sprachdiskrimination ist, neben einer möglichst optimalen Einstellung des CI-Systems, von einer Reihe ätiologischer Faktoren abhängig [2, 14]. Bei individueller bzw. patientenspezifischer gemeinsamer Betrachtung von pHV_KLS_ und pHV_FB_ über den Versorgungszeitraum ließen sich die Effekte eines multidisziplinären CI-Versorgungsprozesses aus Sicht der Autoren elegant darstellen. Die abnehmende Differenz zwischen pHV_KLS_ und pHV_FB_ könnte so als Rehabilitationseffekt interpretiert werden. Zur Untermauerung dieser These könnten weiterführende prospektive Studien die Korrelation der Differenz aus pHV_KLS_ und pHV_FB_ mit subjektiven Qualitätsmaßen aus den Ergebnissen von Fragebögen [1, 13] untersuchen.

Zeh et al. [24] beschrieben den Vorteil stationärer Rehabilitationskonzepte. Dabei analysierten sie unter anderem die Ergebnisse verschiedener audiometrischer Messmethoden jeweils eigenständig und leiteten den Vorteil aus der Zusammenschau der Ergebnisse ab. Die interindividuelle Betrachtung von pHV_KLS_ und pHV_FB_ böte die Möglichkeit, verschiedene Versorgungskonzepte direkt miteinander vergleichbar zu machen. Dabei darf die Zusammensetzung des Patientenkollektivs hinsichtlich limitierender Komorbiditäten [2] keinesfalls vernachlässigt werden. Besonders bei konzeptionellen Umstellungen innerhalb einer CI-versorgenden Einrichtung böte die interindividuelle Betrachtung der pHV über die Zeit eine einfache Möglichkeit, die Wirkung neuer Konzepte zu verdeutlichen.

## Fazit für die Praxis

- Die Betrachtung des prozentualen Hörverlusts erlaubt es, die Ergebnisse von Ton- und Sprachaudiometrie in zwei Variablen zusammenzufassen.
- Die Erhebung aus klinischen Routinedaten ist bei Patienten nach Versorgung mit einem Cochleaimplantat einfach möglich.
- Die Aussagekraft für die Beurteilung der Versorgung mit dem Hörsystem sollte anhand prospektiver Studien mithilfe von patientenzentrierten Instrumenten weiter untersucht werden.
